# Survival from cancer of the pancreas in England and Wales up to 2001

**DOI:** 10.1038/sj.bjc.6604577

**Published:** 2008-09-23

**Authors:** N Starling, D Cunningham

**Affiliations:** 1 Department of Medicine, Royal Marsden Hospital, Downs Road, Sutton, Surrey SM2 5PT, UK

The majority of patients with pancreatic cancer present with advanced disease. Only 10% of patients have potentially operable tumours confined to the pancreas, but, even then, most relapse following surgical intervention. Presenting features include obstructive jaundice, weight loss, lumbar or epigastric pain, dyspepsia, recurrent attacks of pancreatitis and ascites. Paraneoplastic phenomena may occur and a hypercoagulable state may cause thrombotic complications, such as deep vein thrombosis or thrombophlebitis migrans. Smoking and chronic pancreatitis are known risk factors. The relative risk of pancreatic cancer is increased in certain rare familial syndromes, such as Peutz–Jegers syndrome, familial atypical multiple mole melanoma syndrome and hereditary pancreatitis (The European Pancreatic Cancer Research Cooperative (EPCRC, 2004)).

Endoscopic ultrasound (EUS) was introduced in the early 1980s and has assumed a central role in the detection and characterisation of pancreatic tumours. It is particularly sensitive for small tumours and for the assessment of lymph node involvement (Rosch *et al*, 1992). Within the last 10 years, the establishment of EUS guided fine needle aspiration and lymph node sampling has provided a convenient means by which to establish a cytological diagnosis (Chen and Eloubeidi, 2004). Improvements in CT scanning in the 1990s with the development of multiphase thin slice helical CT have enhanced the sensitivity of CT for tumours less than 2 cm (Zeman *et al*, 1997). In practice, CT and EUS will be performed as complementary staging investigations. ^18^F-deoxyglucose-positron emission tomography (FDG-PET), PET-CT and MRI are useful adjuncts with the former particularly useful for the detection of occult metastatic disease. Together, these imaging modalities aim to establish a diagnosis and determine suitability of surgery. Although the detection of operable candidates may improve survival for that group, more accurate imaging is likely to increase the number of patients deemed unresectable and is therefore unlikely to have significantly impacted on survival trends in pancreatic cancer.

In the advanced disease setting, the major therapeutic advance has been the establishment of single agent gemcitabine as a standard of care. This is based on the results of a single randomised clinical trial in 1997 comparing gemcitabine with bolus 5-fluorouracil (5-FU). This demonstrated improved clinical benefit response rates (24 *vs* 5%; *P*=0.0022), median survival duration (5.65 *vs* 4.4 months; *P*=0.0025) 1-year survival (19 *vs* 2%) in favour of gemcitabine (Burris III *et al*, 1997). On the basis of these data, gemcitabine became widely adopted as a first-line palliative therapy in pancreatic cancer following NICE guidance in May 2001, although there may have been some utilisation of this agent before this. The impact of gemcitabine on survival is therefore unlikely to be apparent in the period in question, although the survival benefit for gemcitabine is at best modest.

Surgical resection is offered to a minority of patients. Since 1980 there have been considerable improvements in perioperative morbidity and mortality brought about through advances in surgical techniques, perioperative care and diagnostic imaging. The concentration of surgery within specialist centres with a high volume of cases has also contributed to improved surgical outcomes (Andren-Sandberg and Neoptolemos, 2002). This may not be clearly reflected in the overall survival trends as patients with resectable disease represent a small cohort of the overall pancreatic cancer population. Adjuvant chemotherapy has been developed recently. In the ESPAC-1 trial of observation, chemotherapy (bolus 5-FU) and chemoradiotherapy in 549 patients with resected pancreatic cancer, there was an improvement in survival for the chemotherapy but not the chemoradiotherapy arms. The trial was first reported in 2001 with mature data presented in 2004 (Neoptolemos *et al*, 2001; Neoptolemos *et al*, 2004). A meta-analysis of five adjuvant trials in pancreatic cancer supports the use of adjuvant chemotherapy (Stocken *et al*, 2005). The ongoing phase III ESPAC-3 trial is comparing gemcitabine *vs* bolus 5-FU in the adjuvant setting. Adjuvant therapy would not have influenced survival trends during the period of review, but based on current evidence, many patients now routinely receive postoperative chemotherapy. The integration of radiation therapy incorporating newer techniques for delivery and tumour targeting is currently an expanding area of investigation for patients with both operable and locally advanced disease and for the latter may offer the scope for conversion to resectable disease.

On the basis of the analysis presented, 5-year survival rates have not changed significantly between 1986–1990 and 1996–1999 as might have been predicted from earlier discussion. There have been small improvements in the 1-year survival for both men and women. The improvements in diagnostic imaging and reduction in surgical morbidity/mortality may have contributed to this, although patients with advanced disease would have accounted for the majority of cases. Depending on the uptake of first-line gemcitabine, palliative chemotherapy may account for some of the improvement in 1-year survival. Coordination of patient care through multidisciplinary team meetings established in the late 1990s, may have improved access to relevant services and also impacted on survival trends. The changes in survival among the various deprivation categories have been similarly minimal with a reported slight increase in the deprivation gap in men relative to women at 1 and 5 years.

The survival trends presented are reflective of the aggressive nature of pancreatic cancer. Earlier diagnosis and new therapeutic advances are clearly required to improve this situation. In advanced disease, the routine use of gemcitabine first-line may result in improved survival in the next period of analysis but the magnitude is unlikely to be large. However, for patients fit for therapy, gemcitabine remains a widely used standard of care following several recent negative randomised controlled trials of combination chemotherapy regimens *vs* gemcitabine monotherapy. In a recent meta-analysis a survival benefit was demonstrated for gemcitabine doublets incorporating capecitabine or platinums and this may impact on future practice (Sultana *et al*, 2007).

Significant resources have been applied to elucidating the pathophysiological mechanisms of pancreatic cancer including the role of tumour suppressor and promoter genes and key tumorigenic pathways. Erlotinib, a targeted therapy against the epidermal growth factor pathway (EGFR), has recently been shown in a randomised phase III trial to be superior to gemcitabine alone, again with a modest survival gain (Moore *et al*, 2007). However, preliminary reports of randomised trials of other biological therapies combined in gemcitabine doublets (cetuximab, targeting the EGFR pathway and bevacizumab, targeting the vascular endothelial growth factor axis) have disappointingly not indicated improvement in survival for combination therapy (Kindler *et al*, 2007; Philip *et al*, 2007). To make progress, the impetus therefore remains strong for the continued conduct of high quality preclinical and translational research incorporating newer technologies directed at systems biology, in tandem with the development and evaluation of other rational targeted therapies including the multi-targeted small molecule receptor tyrosine kinase inhibitors and vaccine therapy, likely to be as part of combinatorial therapy.
